# Supplementary material for: Internal Quality Attributes and Sensory Characteristics of ‘Ambrosia’ Apples with Different Dry Matter Content after a Two-Week and a Ten-Week Air Storage at 1 °C
Source: Foods. 2023 Jan 3;12(1):219. doi: 10.3390/foods12010219 (PMC9818383; doi:10.3390/foods12010219)
Supplement: Supplementary file 1 [file foods-12-00219-s001.zip › foods-2085595-supplementary.pdf]

Internal quality attributes and sensory characteristics of 'Ambrosia' apples with different dry matter content after a two-week and a ten-week air storage at 1°C

Supplementary Table S1. Initial pH of the combined juice samples from the apple fruits of each estimated dry matter content (DMC) category prior to titration.

Location	DMC category	Initial pH Average (SE)	
		After 2-week storage	After 10-week storage
**Cawston**	11.5% ± 0.5	4.08 (0.09)	
	12.5% ± 0.5	3.92 (0.07)	3.94 (0.01)
	13.5% ± 0.5	3.95 (0.04)	3.87 (0.03)
	14.5% ± 0.5	3.96 (0.10)	3.85 (0.02)
	15.5% ± 0.5	3.85 (0.13)	
		F(4,11)=0.81, *p*=0.55	F(2,7)=5.15, *p*=0.0498
**Summerland Research and Development Centre (SuRDC)**	13.5% ± 0.5	4.28 (0.03)	
	14.5% ± 0.5	4.08 (0.14)	3.83 (0.03)
	15.5% ± 0.5	4.28 (0.10)	3.94 (0.04)
	16.5% ± 0.5	4.16 (0.02)	3.87 (0.02)
	17.5% ± 0.5	4.16 (0.09)	
		F(4,11)=0.99, *p*=0.45	F(2,7)=3.60, *p*=0.094

Note: Measurements were conducted after 2-week storage and after 10-week storage. Each juice sample consisted 2-3 slices per fruit, of 3 fruits. There were 3 juice samples for each DMC level at each location after a specific storage duration (n = 3). The *p* value showed statistical significance among DMC levels of each location/duration (One-way ANOVA, Tukey's pairwise comparison).

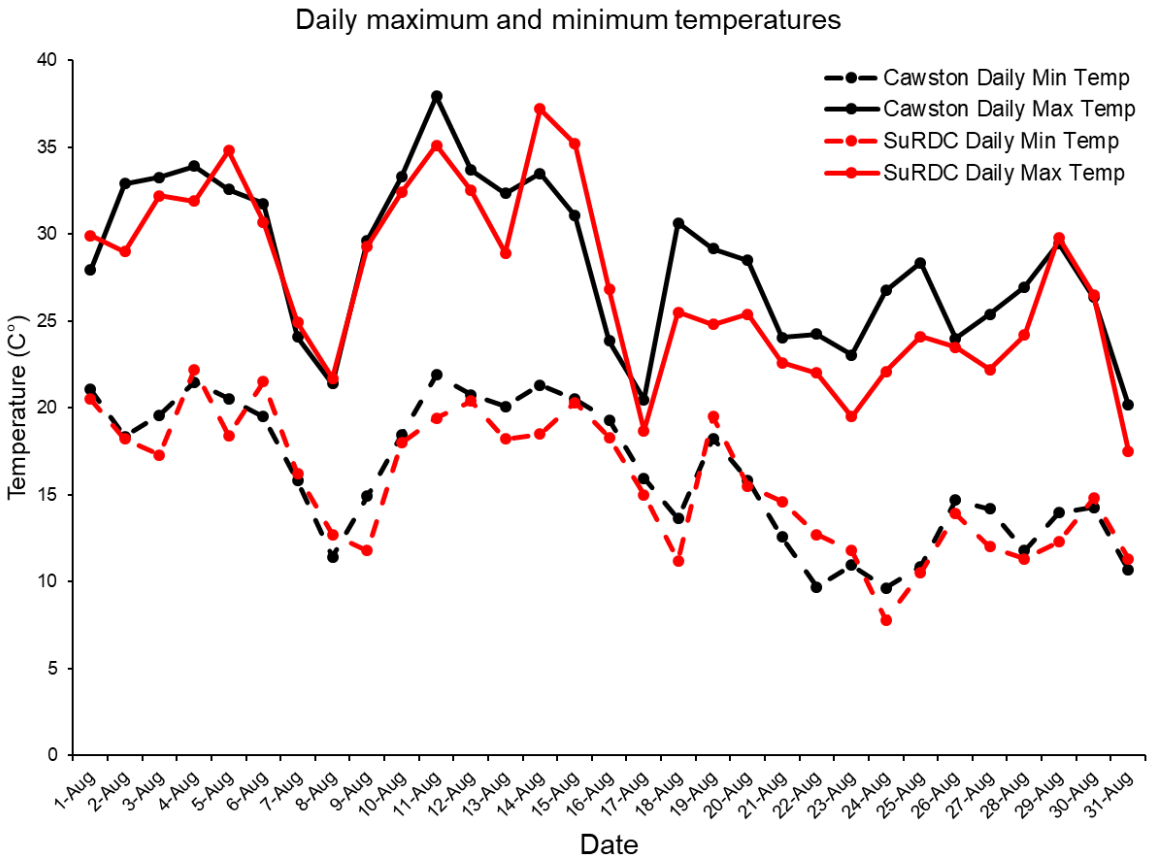

Supplementary Figure S1. Daily maximum and minimum temperatures in August 2021 at two study locations. Data were recorded using HOBO MX2301A sensors.
